# Numerical Simulation of Callus Healing for Optimization of Fracture Fixation Stiffness

**DOI:** 10.1371/journal.pone.0101370

**Published:** 2014-07-03

**Authors:** Malte Steiner, Lutz Claes, Anita Ignatius, Ulrich Simon, Tim Wehner

**Affiliations:** 1 Institute of Orthopaedic Research and Biomechanics, Center of Musculoskeletal Research Ulm, University Hospital Ulm, Ulm, Germany; 2 Scientific Computing Centre Ulm, University of Ulm, Ulm, Germany; Faculdade de Medicina Dentária, Universidade do Porto, Portugal

## Abstract

The stiffness of fracture fixation devices together with musculoskeletal loading defines the mechanical environment within a long bone fracture, and can be quantified by the interfragmentary movement. *In vivo* results suggested that this can have acceleratory or inhibitory influences, depending on direction and magnitude of motion, indicating that some complications in fracture treatment could be avoided by optimizing the fixation stiffness. However, general statements are difficult to make due to the limited number of experimental findings. The aim of this study was therefore to numerically investigate healing outcomes under various combinations of shear and axial fixation stiffness, and to detect the optimal configuration. A calibrated and established numerical model was used to predict fracture healing for numerous combinations of axial and shear fixation stiffness under physiological, superimposed, axial compressive and translational shear loading in sheep. Characteristic maps of healing outcome versus fixation stiffness (axial and shear) were created. The results suggest that delayed healing of 3 mm transversal fracture gaps will occur for highly flexible or very rigid axial fixation, which was corroborated by *in vivo* findings. The optimal fixation stiffness for ovine long bone fractures was predicted to be 1000–2500 N/mm in the axial and >300 N/mm in the shear direction. In summary, an optimized, moderate axial stiffness together with certain shear stiffness enhances fracture healing processes. The negative influence of one improper stiffness can be compensated by adjustment of the stiffness in the other direction.

## Introduction

Fractures typically heal successfully, however five to ten per cent of all fractures show complications such as healing delays or non-unions [1], [2], [3]. These complications are of great clinical relevance due to the large incidence of fractures, which stand as one of the most frequent injuries to the musculoskeletal system [4], [5]. Apart from numerous biological factors [6], [7], local mechanical conditions within a diaphyseal, long bone fracture zone determine the healing course and success decisively [8], [9], [10]. A measure for the mechanical conditions within the fracture site is the interfragmentary movement (IFM), which under physiological loading [11] in fractured human tibiae is highly complex and consists of axial motion, bending, and torsional and translational shear [12], [13].

To stabilize long bone fractures against these loading influences, surgeons use either external fixation, plate fixation, or intramedullary nailing [14]. Each of these different fixation methods show different predominant IFM directions. External and plate fixation lead to predominant axial compressive IFM through bending because of the relatively low bending stiffness of the devices [15]. Intramedullary nails can create remarkable shear movements in the fracture gap caused by the play of the nail within the medullary canal [16].

Based on animal experiments in sheep, it was found that both magnitude and direction of IFM perform important roles; while small and moderate axial compressive IFM are widely accepted to stimulate healing [17], [18], [19], [20], [21], translational shear can delay or inhibit healing processes [22], [23], [24], as do large motion magnitudes in general [25].

This indicates that some of the occuring healing complications might be the result of inhibitive mechanical conditions arising from improper fixation stiffness. Thus, finding an optimal fixation stiffness was aspired by means of sheep experiments [21]. Nevertheless, due to the very limited numbers of *in vivo* experiments and investigated fixation device samples, a general statement of the correlation between fixation stability and healing outcome is hard to achieve from *in vivo* data.

Numerical fracture healing simulation, however, has the abilities to freely define the fixation stiffness independent of the design of the fixation devices used, to define and control the acting loading situation, and to simulate large numbers of arbitrarily defined fixation scenarios. Given a proper corroboration by available and suitable *in vivo* results, *in silico* simulations have large potential to provide insights into the mechanical influence on the healing outcome.

Hence, the aim of the present study was to numerically simulate healing outcomes for sheep diaphyseal fractures under physiological loading, being treated by fixations with various combinations of different axial and translational shear fixation stiffness. Thereby, optimal as well as detrimental configurations of fixation stiffness should be identified.

## Methods

### Numerical fracture healing model

**Figure 1:**
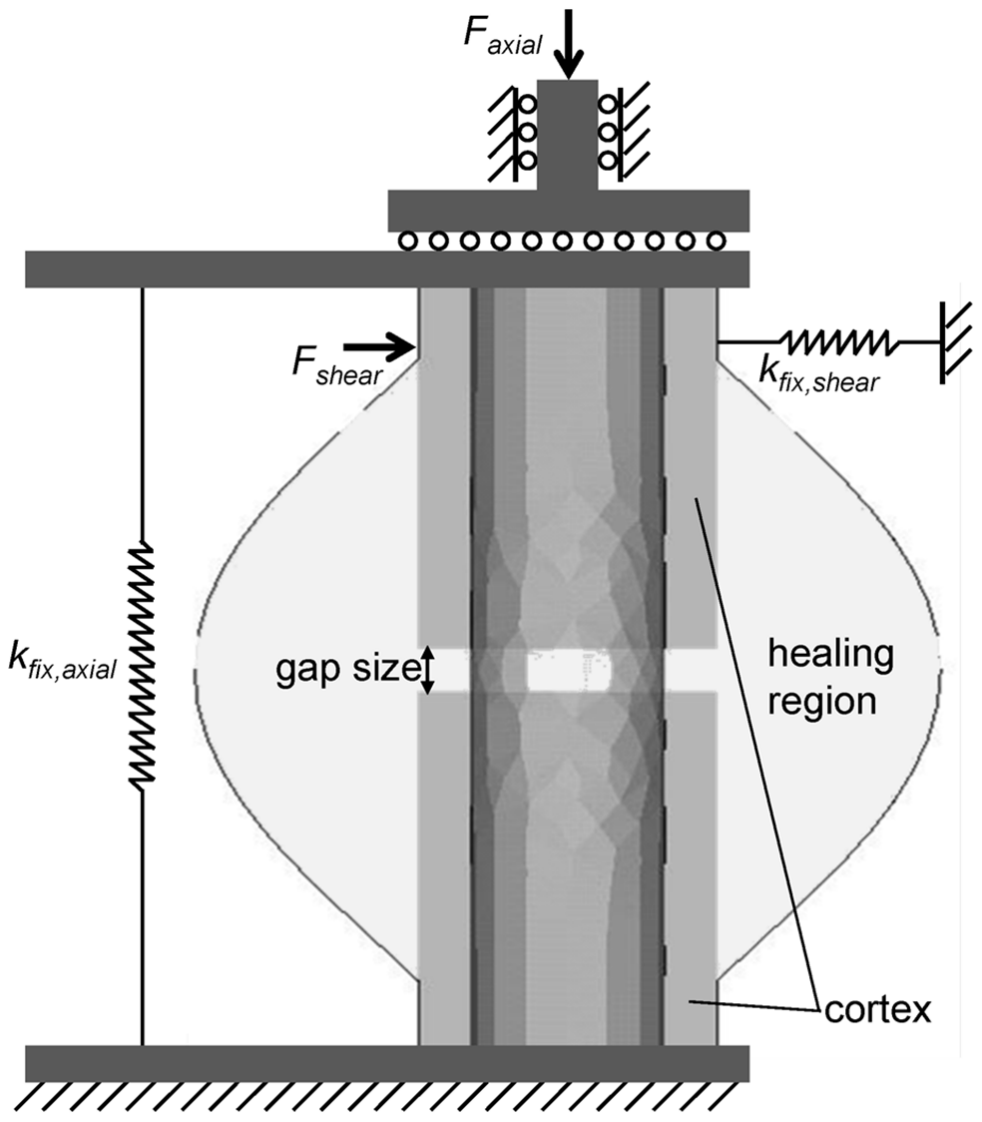
Boundary conditions of the superimposed loading case.

For the present computational study, a three-dimensional finite element model of an idealized mid-diaphyseal, transversal, osteotomy geometry in the ovine tibia (endosteal diameter: 13 mm, periosteal diameter: 20 mm, gap sizes: 1 mm, 3 mm) and its healing region was created and meshed in ANSYS (v14.0, ANSYS Inc., Canonsburg, PA, USA) using tetrahedral elements. Material properties were obtained in a previous study [26] (cf. Table 1) and were assumed as linear elastic and isotropic. Due to large variations in the literature, average loading magnitudes were calculated based on data from three different *in vivo* studies [27], [28], [29] to represent physiological loading conditions in the mid-diaphyseal sheep tibia. Thus, assuming a mean bodyweight of 63 kg [21], an axial compressive load of 840 N and a translational shear load of 200 N, was applied. To represent the use of intramedullary nailing for fracture fixation, the intramedullary (endosteal) healing region was not modeled. Implemented boundary conditions for loading and fixation behavior are shown in Figure 1. Elements within the healing region initially consist of connective tissue that develops into cartilage or bone during the healing process, depending on the local mechanical conditions. This is controlled by a previously published numerical fracture healing simulation algorithm that was described in detail elsewhere [30], [31]. Briefly, the algorithm applies a set of 20 linguistic fuzzy logic rules (Fuzzy Logic Toolbox in MATLAB (v7.11, R2010b), The MathWorks, Inc., Natick, MA, USA) that controls how tissue composition and vascularization for each finite element changes depending on local mechanical and biological stimuli in an iterative process, representing the healing progress. The rules are based on the mechanoregulatory model proposed by Claes and Heigele [32] and represent intramembraneous ossification, chondrogenesis, endochondral ossification, revascularization and tissue destruction. The outputs of this model are the courses of IFM and tissue distribution over the healing time. Additionally, the bending stiffness was calculated for each iteration step by a cantilever bending simulation. The respective bending stiffness *k_Bend_ = EI* is defined as
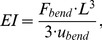
(1)where *L* is the overall length of the bone model, *u_bend_* is the applied displacement, and *F_bend_* is the corresponding reaction bending force. In a previous study [26] the healing algorithm was further calibrated to properly predict fracture healing processes under various loading conditions (particularly under axial compression, torsional and translational shear loading) in sheep. In contrast to previous studies, where a certain initial IFM was allowed by the fixation, in the present work the stiffness of the initial callus material had a remarkable influence. Therefore the existing model was expanded by adding a maturation of the initial connective tissue over the healing time (i.e. a sigmoidal increase from 0.1 MPa, representing fracture hematoma, to 1.4 MPa, representing mature connective tissue, within 8 weeks). Preceding simulations showed that this had no notable influence on predicting our previous calibration load cases [26] properly.

**Table 1:** Material properties of the involved tissues, according to Steiner et al. [26].

	Young’s Modulus,	Poisson’s Ratio,
	*E*_tiss_ in MPa	*ν*_tiss_
Cortical bone	15750	0.325
Woven bone	538	0.33
Fibrocartilage	28	0.3
Connective tissue	1.4	0.33

### Characteristic maps of bending stiffness correlated to fixation stability

To create characteristic maps of healing outcome resulting from fixation stability, healing processes were simulated for a total of 96 different combinations of axial (*k_fix,axial_* = 1, 50, 500, 1000, 1500, 2000, 2500, 3000, 3500, 5000, 7500, 10000 N/mm) and translational shear (*k_fix,shear_* = 1, 100, 200, 300, 400, 500, 600, 1000 N/mm) fixation stiffness for two gap sizes: most in vivo studies in sheep apply gaps of 2–3 mm, therefore a gap size of 3 mm was chosen, additionally, a small gap size of 1 mm was investigated. For each combination, the bending stiffness was reported relatively to the bending stiffness of the respective intact tibial bone (i.e. contralateral side, EI = 100 Nmm^2^) at several healing time points (i.e. 6, 9, and 12 weeks). We used a surface-fitting tool implemented in MATLAB (v7.11, R2010b, The MathWorks, Inc.) to create the continuous characteristic maps from the single data points.

### Extracortical callus volume as predictor of the healing progress

As another indicator for the healing progress, the extracortical bony callus volume was calculated as a percentage of the numerically predefined healing region, where callus formation can take place. This is reported at different healing time points for several exemplary simulations in order to describe the progress of callus development and correlate it to their respective healing success. A further parameter for the callus size is the callus index, which is defined as the ratio between callus and cortex diameters (*CI = diam_callus_*/*diam_cortex_*).

### Corroboration on literature data of different fixation stiffness

For comparison of the created characteristic maps with experimental data, the literature regarding studies which investigated the influence of mechanics on fracture healing in sheep was reviewed. Experimental conditions of the reviewed studies needed to strongly agree with the present simulation parameters. Thus, *in vivo* cases with osteotomies in ovine long bone diaphyses with appropriate gap sizes were chosen. Studies which did not properly characterize the stiffness of the fixation device could not be included. Additionally, only fixations with steady, linear stiffness behavior could be included, and therefore study results where a non-linear fixation stiffness was used (e.g. very rigid fixation allows a certain amount of initial IFM by very low fixation stiffness, cf. Claes et al. [10]) were not taken into account. Furthermore, IFM should only result from the stiffness behavior of the bone-implant construct passively under physiological loading; active IFM applied by actuators could not be included in this study.

## Results

### Characteristic maps of bending stiffness correlated to fixation stability

**Figure 2:**
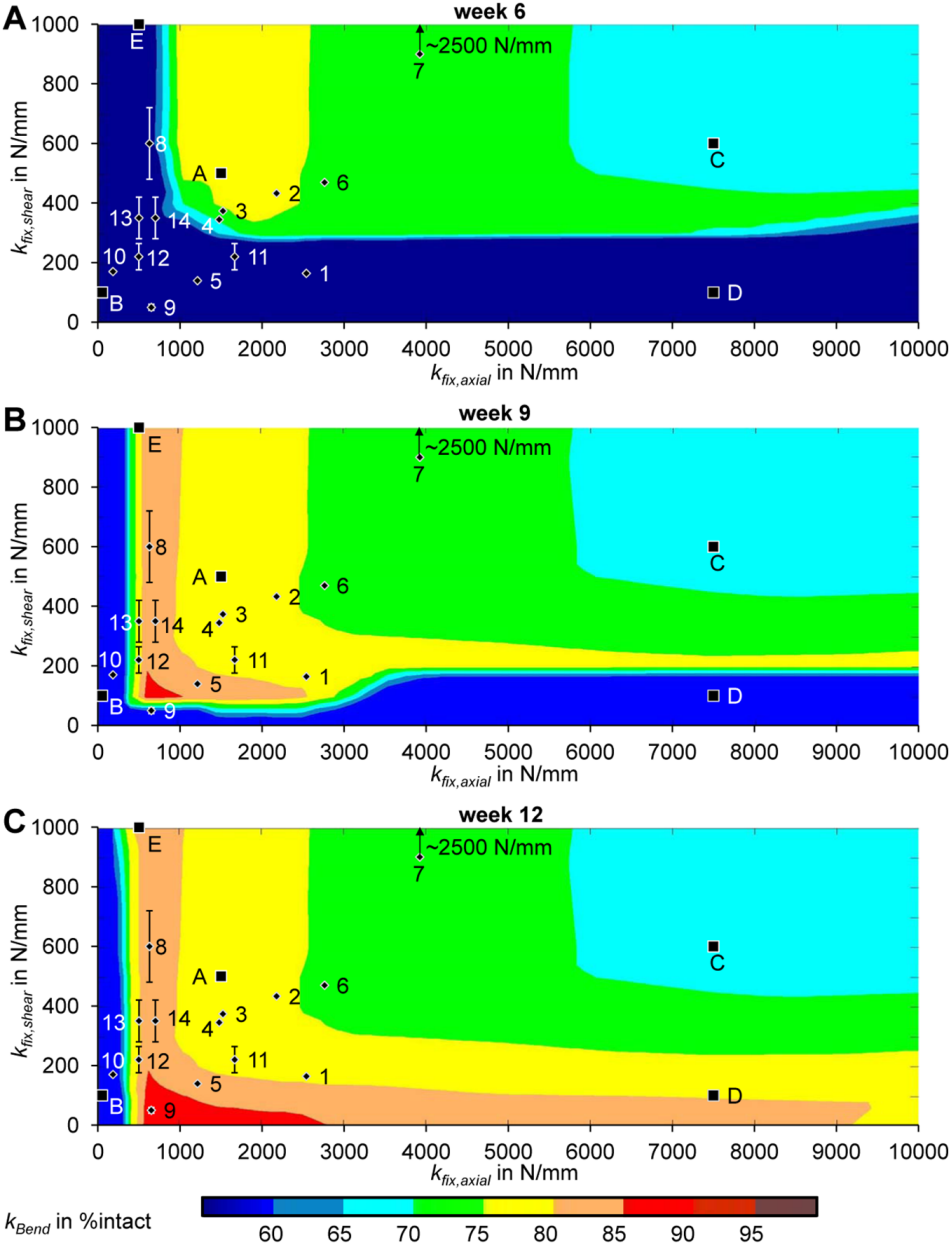
3 mm osteotomy: characteristic maps of bending stiffness depending on the fracture fixation stiffness in axial (k_fix,axial_) and shear (k_fix,shear_) direction after A 6 weeks of healing B 9 weeks of healing C 12 weeks of healing. Bending stiffness (k_Bend_) is given as the percentage of the intact (contralateral) bone bending stiffness. Numbered data points refer to experimental data in Table 3, error bars indicate estimated values (20% error) for unknown shear stiffness of the devices. Letters indicate positions of the exemplary simulation results in Figure 5.

The bending stiffness depends on the fixation stiffness in both the shear and axial loading directions as shown in Figure 2 at three healing time points for the physiological loading of an ovine, diaphyseal fracture with 3 mm gap size. At week 6 (Figure 2A), cases with 1000 N/mm−2500 N/mm axial stiffness combined with stiffness greater than 300 N/mm in shear direction are bridged with a resulting bending stiffness greater than 75% of the respective intact bone. Cases with axial stiffness larger than 3000 N/mm also show bridging but develop a bending stiffness smaller than 75%. A large proportion of cases still show no bridging (i.e. black area). At week 9 (Figure 2B), cases with axial stiffness of 500 N/mm–1000 N/mm show bridging with large callus development, leading to high bending stiffness, greater than 80%, even for more flexible shear stiffness (>100 N/mm). No healing is observed for overly flexible fixation in both stiffness directions. At week 12 (Figure 2C), only cases which are overly flexible in the axial direction continue to show non-unions. Cases with small shear rigidity developed large calluses, which lead to bending stiffness greater than 80%.

**Figure 3:**
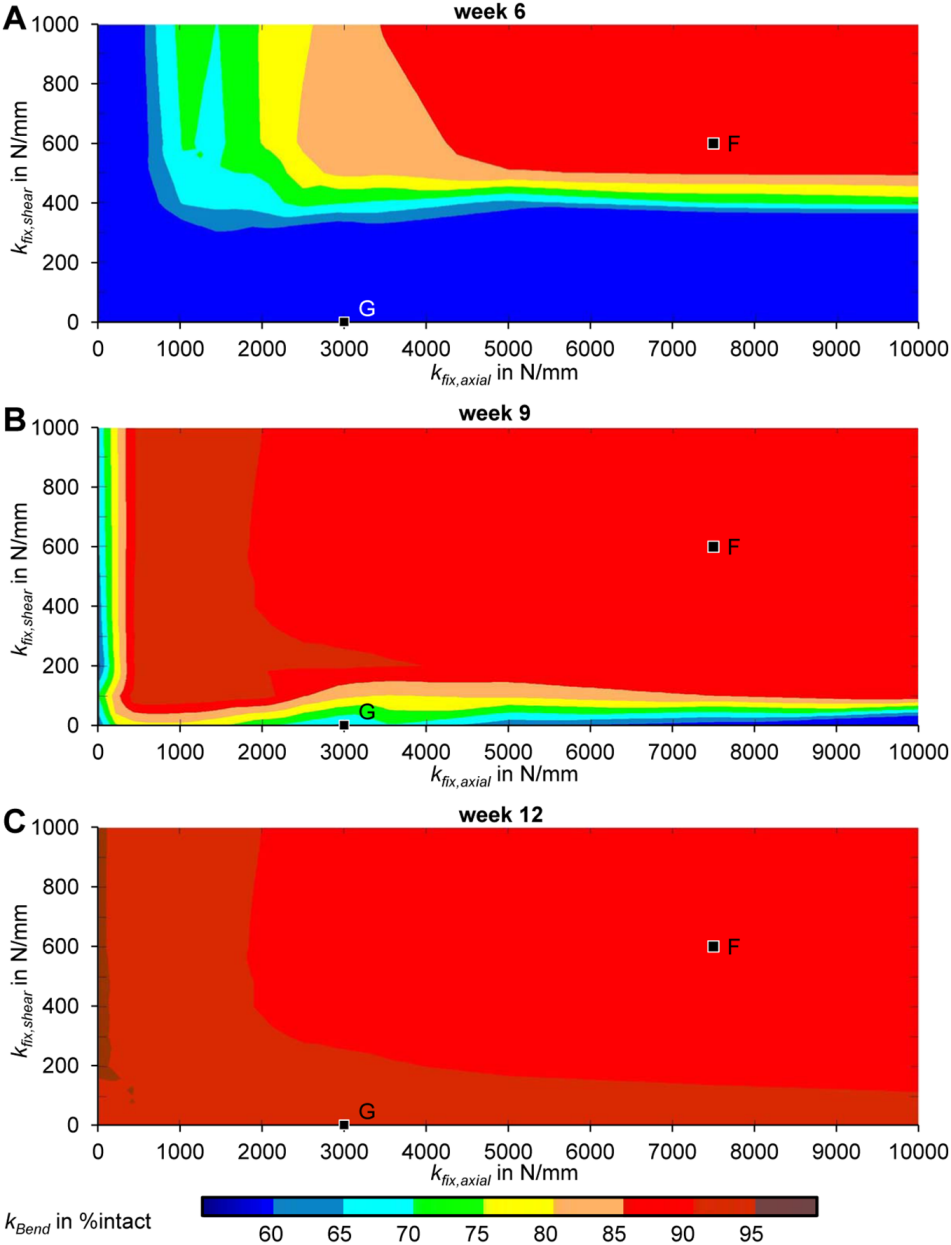
1 mm osteotomy: characteristic maps of bending stiffness depending on the fracture fixation stiffness in axial (k_fix,axial_) and shear (k_fix,shear_) direction after A 6 weeks of healing B 9 weeks of healing C 12 weeks of healing. Bending stiffness (k_Bend_) is given as the percentage of the intact (contralateral) bone bending stiffness. Letters indicate positions of the exemplary simulation results in Figure 6.

Bending stiffness for the same fixation stabilities but for a small gap size of 1 mm show less impact of the fixation stability as compared to a medium gap size of 3 mm. At week 6 (Figure 3A), a large proportion of cases with large stiffness in both directions (i.e. >400 N/mm in shear; and >2500 N/mm in axial direction) already show healing with large bending stiffness greater than 80%. At week 9 (Figure 3B), only cases with very flexible stiffness in both directions still show non-unions. However, these are healed at 12 weeks (Figure 3C) and demonstrate very large resulting bending stiffness (>90%).

**Figure 4:**
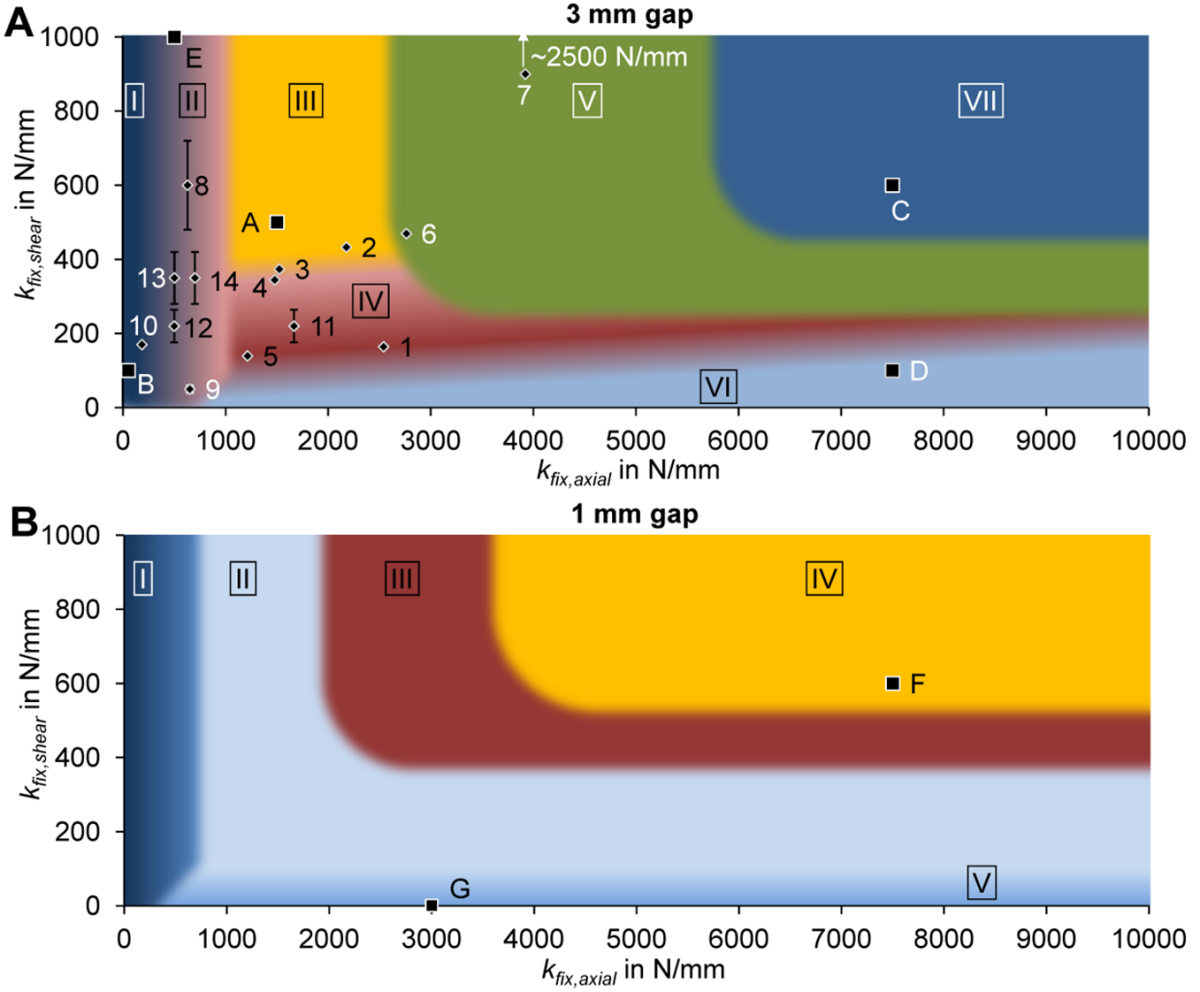
Qualitative characteristic maps of healing outcome depending on the fracture fixation stiffness in axial (k_fix,axial_) and shear (k_fix,shear_) direction for A 3 mm fracture gap; B 1 mm fracture gap. Roman numerals refer to areas of different healing outcomes as explained in detail in Table 3. Letters indicate positions of the exemplary simulation results in Figures 5 and 6.

**Figure 5:**
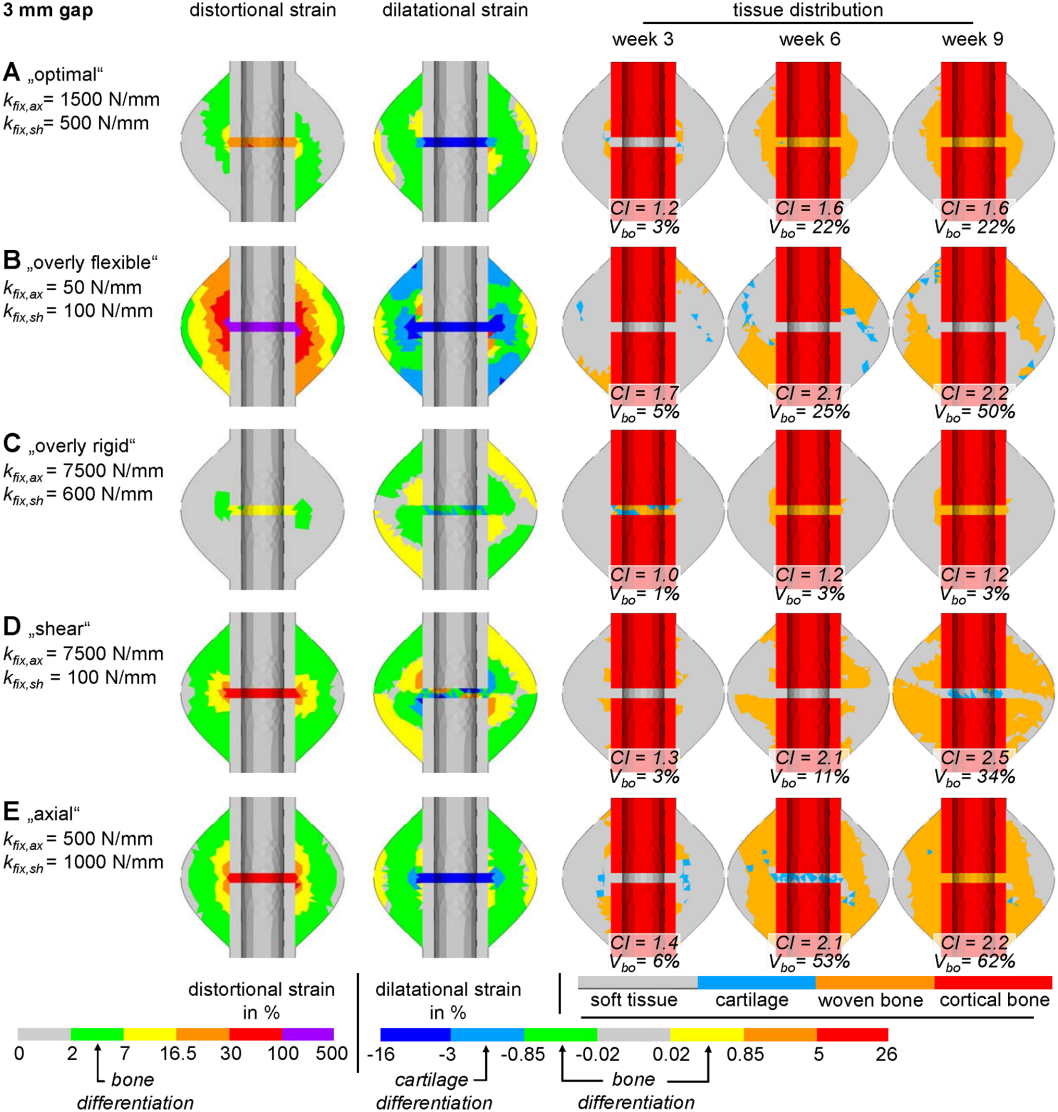
Five different exemplary simulations for a 3 mm gap size. For each case the initial distortional and dilatational strain field is shown, which determine the tissue differentiation following the hypothetic rules of Claes and Heigele [32]. Respective tissue stimulating strain ranges are indicated at the color bars. Additionally, the tissue distribution, as well as the percentage of extracortical bony callus volume (V_bo_), and the callus index (CI) at 3, 6, and 9 weeks of healing are displayed for **A** optimal fracture fixation; **B** overly flexible fixation leading to non-union; **C** overly rigid fixation leading to inhibition of callus development with unstable bending stiffness; **D** a predominant shear load case; **E** a predominant axial load case. Letters are according to diagrams in Figures 2 and 4.

Qualitatively, the characteristic map for the 3 mm fracture gap can be classified into seven different regions according to Figure 4A and Table 2. Hence, the optimal range (region III, light grey) is 1000 N/mm−2500 N/mm axial stiffness, combined with stiffness greater than 300 N/mm in shear direction (cf. Figure 5, case A). Overly flexible fixation in either direction (approximately <200 N/mm in axial and <100 N/mm in shear direction) results in delayed healing due to instability and high tissue strains (cf. Figure 5, case B). Impeding influences of low shear stiffness can be compensated by adjusting the axial stiffness; vice versa, this compensatory effect is limited (i.e. increased shear stiffness does not improve the negative effect of low axial stiffness). Furthermore, very high axial stiffness (approximately >3000 N/mm) of the fixation device also results in delayed healing (cf. Figure 5, case C). This effect can, in turn, be partly compensated by decreasing the shear stiffness to approximately 200 N/mm. Furthermore, cases with predominant shear movements (i.e. large axial rigidity, low shear stiffness, cf. Figure 5, case D) show delayed healing when compared against cases with predominant axial movements (cf. Figure 5, case E).

**Table 2:** Comments on the qualitative characteristic maps in Figure 4.

	3 mm gap	1 mm gap
I	**Non-union** due to overly flexible fixation	**Delayed healing** with large callus formationand high bending stiffness (>95%) after 12 weeks
II	**Slightly delayed healing** with largecallus formation and high bendingstiffness (>80%) after 9 weeks	**Slightly delayed healing** with large callusformation and high bending stiffness (>90%)after 9 weeks
III	**Optimal healing** with fast formation ofmoderate callus volume showing a highbending stiffness (>75%) after 6 weeks	**Quick healing** with moderate callus formationshowing sufficient bending stiffness (>85%)after 6 weeks
IV	**Slightly delayed healing** with moderatecallus formation showing sufficient bendingstiffness (>75%) after 9 weeks	**Optimal healing** - fast formation of moderatecallus volume showing a high bending stiffness(>90%) after 6 weeks
V	**Suboptimal healing** - overly rigid fixationstiffness shows rapid but small callusformation resulting in insufficient bendingstiffness (kB<75%) at 6 weeks, which does notincrease further	**Delayed healing** with large callus formationand high bending stiffness (>90%) after 12 weeks
VI	**Delayed healing** with large callus formationand high bending stiffness (>85%)after 12 weeks	–
VII	**Unfavorable healing** - overly rigid fixationstiffness shows rapid but very small callusformation resulting in unstable bendingstiffness (kB<70%) at 6 weeks, whichdoes not increase further	–

**Figure 6:**
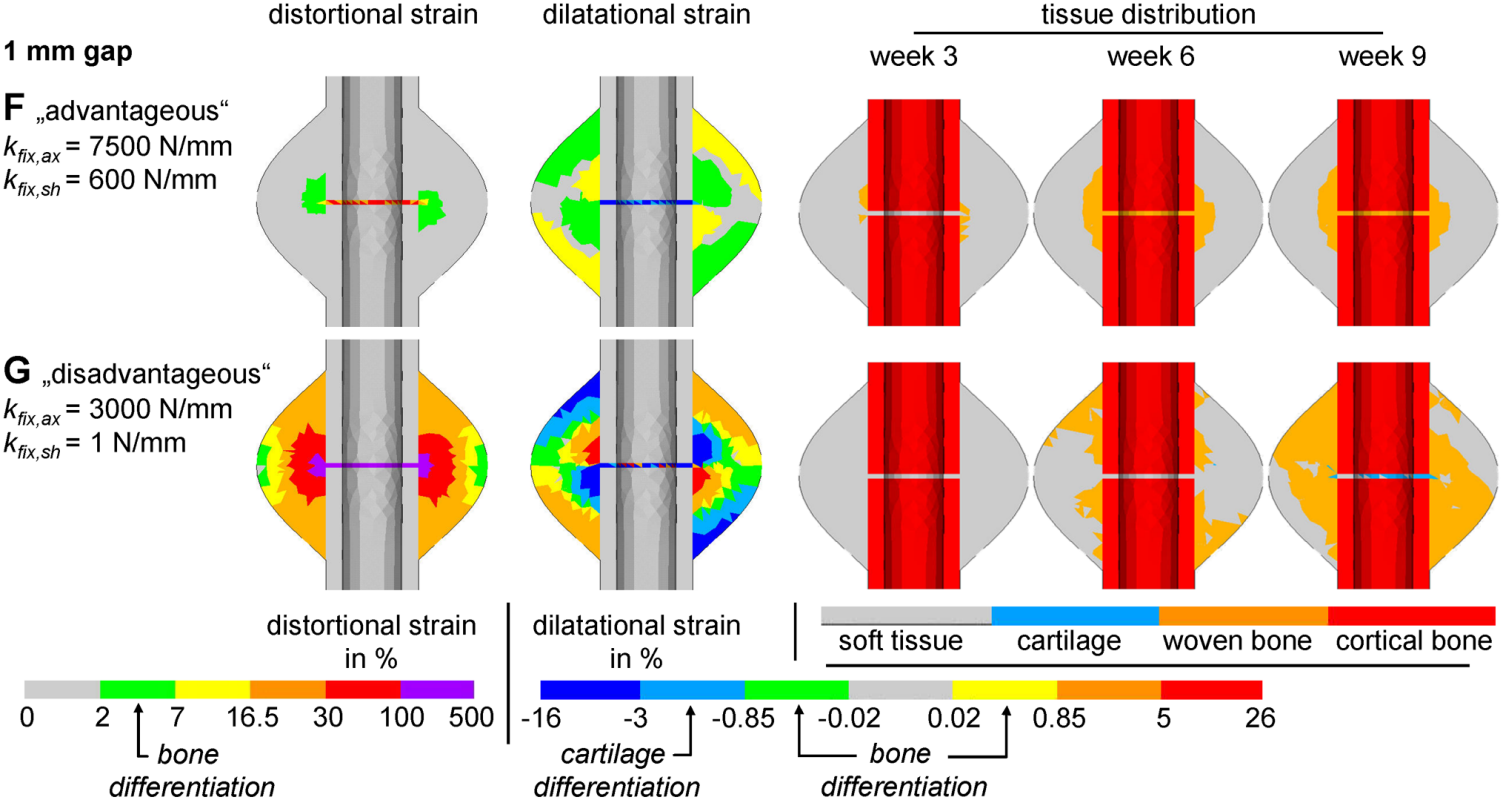
Exemplary simulations for a 1 mm gap size. For each case the initial distortional and dilatational strain field is shown, which determine the tissue differentiation following the hypothetic rules of Claes and Heigele [32]. Respective tissue stimulating strain ranges are indicated at the color bars. Additionally, the tissue distribution at 3, 6, and 9 weeks of healing are displayed for **F** advantageous fixation; **G** disadvantageous (overly flexible) fixation. Letters are according to diagrams in Figures 3 and 4.

The findings for the 3 mm fracture gap were qualitatively classified into five different categories according to Figure 4B and Table 2. Thus, the best healing results are obtained for very high fixation rigidity (cf. Figure 6, case F), due to a direct gap ossification healing mechanism. For more flexible fixations, healing is delayed compared to the 3 mm gap results. However, those delayed healing cases do not result in non-unions (cf. Figure 6, case G).

### Extracortical callus volume as predictor of the healing progress

As another measure of the healing outcome, the relative extracortical callus volume was investigated for exemplary cases A–E with a gap size of 3 mm. This reveals that the optimal stiffness configuration (cf. Figure 5, case A) leads to only moderate callus formation of 20–30% (CI = ∼1.6). Overly flexible configurations lead to extracortical, bony callus formation of less than 50%, which does not result in bony bridging (Figure 5, case B). Likewise, for overly rigid configurations no extracortical bony callus is developed (>4000 N/mm axial stiffness combined with >400 N/mm shear stiffness leads to <10% extracortical bony callus). In a majority of these cases, bony bridging occurs early in the healing process, but only intercortically with minimal extracortical bone formation (cf. Figure 5, case C).

The largest CI is shown in case B at week 3, which however results in non-union after 9 weeks, whereas case A shows optimal healing (within 5–6 weeks) although it has developed only a moderate CI of 1.6 after 6 weeks. Furthermore, the CI is not directly correlated to the actual callus size, since it only accounts for the largest callus diameter, but not for the total callus volume. This is visible when comparing cases D and E in Figure 5, which both show a large CI of 2.5, and 2.2 after 9 weeks, respectively but a callus volume which differs by a factor of 2.

### Corroboration on literature data of different fixation stiffness

Review of the literature on fracture healing experiments in sheep revealed only a limited number of studies which could be applied to corroborate the obtained computational results, as listed in Table 3. Only experiments which applied 2–3 mm osteotomies could be included and were assigned to the simulations of 3 mm gap size. Moreover, only few studies reported values for the stiffness of their fixation devices in both, axial as well as shear direction. Thus, also cases were included where only the axial stiffness was reported. For these, ranges of shear stiffness (20% - error bars in Figures 2 and 4) were estimated based on comparable fixation devices. This data acts as orientation to classify existing fixation methods within the characteristic map presented, and to evaluate the validity of the numerical predictions. Due to the variety of different criteria of measuring healing outcomes in the included studies, a direct quantitative comparison between them and to the present results could not be realized. Qualitatively, the predictions are generally in good accordance to experimental results. The best healing outcomes are predicted for cases 2, 3, and 4, which were also experimentally classified [21] as “good” or “excellent healing” (i.e. torsional moment at failure after 9 weeks between 66.3% and 83% of contra-lateral intact tibia, cf. Table 3). Desirable healing outcomes are also predicted for case 8, as confirmed by the respective experiments [33], [34]. Slightly delayed but still acceptable healing times are predicted for cases 1, 5, 12, 13, and 14, which does not conflict with the *in vivo* findings [21], [34], [35]. However, when compared to case 13, the simulations predicted an advanced healing for case 14, which is in contrast to the experiment where a greater healing delay was observed for case 14 [34]. Delayed healing is simulated for cases 6 and 9. For case 6, this is in acceptable agreement with the *in vivo* findings [21] whereas for case 9 experimental results show continuation of non-unions after 6 months [25] while the simulation suggests healing at around 10 weeks. The worst healing is predicted for cases 7 and 10. This was experimentally verified for case 7 [33], whereas the simulation for case 10 predicts worse healing than experimentally detected [36]. In general, but especially for cases 3, 4, 5 and 11, our model tends to predict slightly faster healing than experimentally measured [21], [37].

**Table 3:** Literature data of numerous experiments, investigating the healing outcome under different fixation devices on osteotomies in long bone diaphyses of sheep.

#	Fixation device	Healing outcome	Axial stiffnessin N/mm	Shear stiffnessin N/mm
1	Medially mountedmonolateral externalfixator [21]	Torsional moment at failureafter 9 weeks: 61.5% ofcontra-lateral intact tibia	2540	164
2	Anteromedially mountedmonolateral externalfixator [21]	Torsional moment at failureafter 9 weeks: 83% ofcontra-lateral intact tibia	2177	433
3	Rigid monolateralexternal fixator [21]	Torsional moment at failureafter 9 weeks: 68.2% ofcontra-lateral intact tibia	1523	374
4	Semirigid monolateralexternal fixator [21]	Torsional moment at failureafter 9 weeks: 66.3% ofcontra-lateral intact tibia	1479	344
5	Unreamed tibial nail [21]	Torsional moment at failureafter 9 weeks: 52.8% ofcontra-lateral intact tibia	1213	139
6	Angle-stable tibialnail [21]	Torsional moment at failureafter 9 weeks: 64.1% ofcontra-lateral intact tibia	2762	469
7	Locked plating [33]	Torsional strength after 9weeks: ∼42% of contra-lateral intact tibia	3922*	2500*
8	Far cortical locked plating [33]	Torsional strength after 9weeks: ∼67% of contra-lateral intact tibia	628**	600**
9	Mechanically criticalexternal fixator [25]	Torsional moment at failureafter 9 weeks: 14% of contra-lateral intact tibia	650	50***
10	Unilateral externalfixator [36]	Bending stiffness after6 weeks: 60% of contra-lateral intact tibia	183	170
11	Rigid unilateral external fixator/actuator [37] (2 mm gap)	Bending stiffness after6 weeks: 24% of contra-lateralintact tibia	1666	220***
12	Rigid unilateral externalfixator/actuator [35](2.6 mm gap)	Bending stiffness after 8 weeks: 60–69%of contra-lateral intact tibia	498	220***
13	Monolateral externalfixator [34], 35 mmpin offset	Week 6: advanced healing,week 12: bony bridging	500	350***
14	Monolateral externalfixator [34], 25 mmpin offset	Week 6: less advanced healing,week 12: bony bridging	700	350***

*shear and axial stiffness numerically calculated (FE-model) – shear stiffness exceeds characteristic map and is marked by an arrow.

**axial stiffness prior to bony contact of the pins, shear stiffness estimated with 20% error.

***shear stiffness assumed based on comparable devices −20% error estimated.

## Discussion

Besides systemic, biological factors, the mechanical conditions within a fracture gap influence the bone healing process decisively. The present study focuses on these mechanical effects. Thus, characteristic maps were created which show the bending stiffness of an osteotomized sheep tibia as a function of superimposed translational shear and axial compressive fixation stiffness at several healing time points under physiological loading conditions. To evaluate the validity of the obtained simulation results, numerous outcomes of appropriate experiments were compared to the generated characteristic maps.

The findings presented go beyond the *in vivo* experimental conclusions of Epari et al. [21], who found that enhanced healing outcomes can be achieved especially through optimization of the axial stiffness to moderate values and limitation of the translational shear flexibility. Our results confirm these findings, and furthermore show that both loading directions are able to accelerate as well as delay healing. Positive or negative influences of one directional stiffness can be diminished by adjusting the stiffness of the other direction appropriately. Our results suggest that positive healing effects due to appropriate axial stiffness can only be impaired to a limited extent by disadvantageous shear stiffness. Vice versa, negative impacts of too low of a shear stiffness can clearly be compensated by favorable axial stiffness. Due to the small magnitudes of loading in shear direction and superimposed influences of axial loading, these results are not in contrast to our previous findings [24], which stated that, under equal mechanical conditions, isolated shear movements are more detrimental for fracture healing processes than isolated axial compressive movements. Because of the limited number of appropriate *in vivo* studies, our findings could only be corroborated for 3 mm gap situations, nonetheless we also investigated a small gap size of 1 mm. We found that small gaps result in desirable healing almost independently of the stiffness configuration. We assume that this is the effect of direct gap ossification, as was found in other experiments applying minimal IFM on small defects in sheep [18], [38]. However, for more flexible fixations, healing is delayed compared to the 3 mm gap results, which is due to the larger strains that arise from the small gap size being exposed to the same loading conditions as the larger 3 mm gap. Regarding the effects of different fixation devices, the present characteristic maps suggest ranges of fixation stiffness in axial and shear direction with the best healing outcome, which are most closely reached by external fixators. Tibial nails or internal plates in their current designs are found to be less stimulatory due to their large rigidity in axial direction [39], [40]. To overcome these negative influences for internal plates, a far cortical locking method was developed, which shifts the axial stiffness to that of external fixators leading to more favorable healing success in sheep experiments [33], [41]. Another approach was the development of dynamic locking screws, which also increased axial motion especially at the near-plate cortex [42], [43]. For intramedullary nails this problem was faced by decreasing their axial stiffness to achieve accelerated healing without changing the torsional or translational shear rigidity of the nails [40]. However, intramedullary nails are widely used and show good clinical outcomes in general [44]. As explanation, we assume that sufficient stimulatory axial IFM is created by the relatively low bending rigidity of intramedullary nails [21], [45].

The characteristic maps presented indicate areas where mechanical stimulation is insufficient for callus formation for the 3 mm gap. According to Perren et al. [46], this effect can be regarded as primary bone healing under very stable conditions. Our simulations predict the development of very small woven bone callus around the gap (Figure 5, case C), which provides no sufficient stabilization of the fracture and might lead to re-fractures. This is expressed by the low bending stiffness arising from the small callus area moment of inertia and the low material stiffness of the new developed woven bone tissue (cf. Table 1). These findings are confirmed by clinical studies which report re-fractures after the removal of very rigid osteosynthesis devices, especially of internal plates in the forearm [47], femur [48], clavicle, and tibia [49], even though they had been classified as “successfully healed” before removal. Therefore, these very rigid cases lead to unfavorable healing outcomes under physiological conditions.

An examination on the ability of the callus size (CI or callus volume) to serve as an indicator for the success of fracture healing revealed that no dependable prognosis of the healing outcome can be made solely based on the callus size at early healing time points. This is according to Marsh [50], who stated that stiffness measurement is more appropriate than the CI to predict functional outcome of fracture healing in patients. Furthermore, neither the CI nor the callus volume account for the shape of the callus, which can be symmetrical under axial dominated IFM or asymmetrical under shear dominated IFM. The latter show smaller total callus volume, which still has the ability to bridge in the far periphery, resulting in delayed healing compared to axially dominated cases with symmetrical callus development (cf. case D versus case E in Figure 5).

The results of this study show that the optimal healing outcome is reached with a moderate callus volume. For very small calluses, we predict unstable healing due to insufficient mechanical stimulation, whereas large calluses can result in healing, delayed healing or even non-unions despite the large callus volume. The latter can be characterized as hypertrophic non-unions, where large calluses are developed which will not result in bony bridging due to large persisting IFM.

There are two major drawbacks which influence the clinical relevance of the present study: (1) it is a numerical study which is accompanied with several modeling assumptions and limitations. First, we excluded endosteal healing regions; this was reasonable since it represents the situation for intramedullary nailing, and pre-investigations indicated that it has no remarkable effect on the simulated healing results. Furthermore, revascularization after fracture is mainly derived rather from surrounding soft tissue and periosteum than from the bone marrow [51], [52], [53]. Second, we used a linear description for our callus model which includes linear elastic and isotropic material properties as well as a linear behavior of the fixation device. Third, we applied a simplified, superimposed, and averaged loading scenario, consisting of axial compressive and translational shear loading. This represents the physiological conditions at the fracture site in sheep only to a limited extent since additionally axial distraction, bending, and torsional shear occurs [27], [28], [29] and forces for other activities than normal walking (i.e. running, jumping, and short impact forces) are not known and could not be considered. From a mechanical point of view, it is reasonable to focus on axial compression and translational shear, which represent the predominant loading directions within a fracture site since bending mainly produces axial compression within the fracture gap and stiff fixation devices such as intramedullary nails produce shear loading due to play within the medullary canal [16]. Despite these model limitations, our study uses the advantages of numerical simulation to deliver unprecedented, continuous data maps of fixation stiffness and their resulting healing outcomes, by extending results from previous *in vivo* experiments, which themselves provide only punctual information due to their limited number. Furthermore, the corroboration of the numerical simulations depends on the availability of adequate *in vivo* data, which is still limited. Although for numerous cases the respective shear stiffness could only be estimated and was assigned with 20%-error bars, the underlying algorithm was calibrated on various loading conditions in sheep [26] and the characteristic maps of the present study were corroborated by several suitable *in vivo* data points. Thereby, the applied model reaches a relatively high validity to properly predict fracture healing processes in sheep.

(2) This study simulated the fracture healing processes in sheep. Apart from differences between sheep and humans in the loading situation [11], [12], [13] and the fracture geometry, our results are not directly transferable to the human situation because, clinically, large variations of different fracture types and circumstances occur. However, our results can approximately be extrapolated to the clinical situation by comparing our results for idealized sheep fractures to simple, transverse tibia fractures in patients without additional injuries and diseases. Thus, Marsh [50] reports rapid healing in patients to occur after 10 weeks, whereas delayed healing takes around 20 weeks. This was also found by Claes et al. [54], who observed healing for simple, closed, Type A fractures in patients after 10 weeks; for more severe and complex fractures, delayed healing takes around 15–20 weeks. With this quantitative data, we assume as an extrapolation factor that fracture healing in sheep is approximately 1.7–1.8 times faster than in human patients. This extrapolation to the human situation can serve as orientation for fracture care optimization. Furthermore, these results could be helpful for the interpretation of experimental findings on fracture healing, when different fixation devices (e.g. intramedullary nails vs. external fixation) were used.

In summary, this study was able to simulate the influence of fixation stiffness on fracture healing processes and revealed the optimal fixation stiffness configuration for rapid fracture healing. The presented findings provide numerous insights into desirable or disadvantageous mechanical conditions which help to optimize fracture treatment by adjustment of the fracture fixation stability in both axial and shear directions. We conclude that an optimized, moderate axial stiffness together with certain shear rigidity is essential for enhanced fracture healing. Furthermore, inhibiting influences of one loading direction can be compensated by adjusting the other directional stiffness appropriately.
